# An Image-Based Workflow for Objective Vessel Wall Enhancement Quantification in Intracranial Aneurysms

**DOI:** 10.3390/diagnostics11101742

**Published:** 2021-09-22

**Authors:** Sricharan S. Veeturi, Nandor K. Pinter, Andre Monteiro, Ammad A. Baig, Hamid H. Rai, Muhammad Waqas, Adnan H. Siddiqui, Hamidreza Rajabzadeh-Oghaz, Vincent M. Tutino

**Affiliations:** 1 Canon Stroke and Vascular Research Center, Buffalo, NY 14203, USA; sanyasis@buffalo.edu (S.S.V.); mwaqas@ubns.com (M.W.); asiddiqui@ubns.com (A.H.S.); hrajabza@buffalo.edu (H.R.-O.); 2 Department of Mechanical and Aerospace Engineering, University at Buffalo, Buffalo, NY 14260, USA; 3 Department of Neurosurgery, University at Buffalo, Buffalo, NY 14203, USA; npinter@dentinstitute.com (N.K.P.); amonteiro@ubns.com (A.M.); abaig@ubns.com (A.A.B.); hrai@ubns.com (H.H.R.); 4 Dent Neurologic Institute, Buffalo, NY 14226, USA; 5 Department of Pathology and Anatomical Sciences, University at Buffalo, Buffalo, NY 14203, USA

**Keywords:** vessel wall enhancement, T1 non-enhanced MRI, contrast-enhanced MRI, intracranial aneurysms, semi-automated, rupture risk

## Abstract

Background: VWE in contrast-enhanced magnetic resonance imaging (MRI) is a potential biomarker for the evaluation of IA. The common practice to identify IAs with VWE is mainly based on a visual inspection of MR images, which is subject to errors and inconsistencies. Here, we develop and validate a tool for the visualization, quantification and objective identification of regions with VWE. Methods: N = 41 3D T1-MRI and 3D TOF-MRA IA images from 38 patients were obtained and co-registered. A contrast-enhanced MRI was normalized by the enhancement intensity of the pituitary stalk and signal intensities were mapped onto the surface of IA models generated from segmented MRA. N = 30 IAs were used to identify the optimal signal intensity value to distinguish the enhancing and non-enhancing regions (marked by an experienced neuroradiologist). The remaining IAs (n = 11) were used to validate the threshold. We tested if the enhancement area ratio (EAR—ratio of the enhancing area to the IA surface-area) could identify high risk aneurysms as identified by the ISUIA clinical score. Results: A normalized intensity of 0.276 was the optimal threshold to delineate enhancing regions, with a validation accuracy of 81.7%. In comparing the overlap between the identified enhancement regions against those marked by the neuroradiologist, our method had a dice coefficient of 71.1%. An EAR of 23% was able to discriminate high-risk cases with an AUC of 0.7. Conclusions: We developed and validated a pipeline for the visualization and objective identification of VWE regions that could potentially help evaluation of IAs become more reliable and consistent.

## 1. Introduction

Vessel wall enhancement (VWE) has emerged as a potential image-based biomarker for the assessment of intracranial aneurysm (IA) rupture risk. VWE is a phenomenon that is observed in contrast-enhanced magnetic resonance imaging (MRI), in which the IA wall exhibits a distinctly higher intensity compared to the non-enhanced MR image. Clinical studies have reported that unstable and rupture-prone aneurysms are more likely to demonstrate enhancement features compared to those that remain stable, indicating that VWE may be a viable biomarker to delineate high-risk lesions [1,2,3]. In addition, enhancing IAs may reflect clinically important pathobiology, as several studies [4,5,6,7] correlated such phenomenon with inflammatory cell infiltrates and other histopathological changes associated with IA progression. 

Currently, identifying whether an aneurysm is demonstrating VWE can be challenging. In clinical practice, this is primarily conducted via a visual inspection of non-enhanced and contrast-enhanced T1-weighted MR images, after which the type (circumferential or focal) and intensity (strong, faint or none) of the enhancement can be classified. VWE assessment in this manner is not reproducible and is susceptible to bias and human error. Therefore, an objective pipeline for the 3D mapping and visualizing of VWE and the delineation of enhancing regions is required for an objective and reliable evaluation of IAs. However, no such tool exists. Up until now, only manual marking of VWE regions or projected MRI intensities on 3D segmented IA geometries have been demonstrated, both of which carry high potential for errors due to difficulty in accurately co-localizing data to the segmented angiographic image [8,9,10,11]. 

In this study, we aimed to develop a semi-automated pipeline for the 3D visualization and objective quantification of VWE regions on IAs. To this end, we collected and co-registered time of flight (TOF) magnetic resonance angiography (MRA) images and high resolution non-enhanced and contrast-enhanced MR images from patients with unruptured IAs. We then created a method to map normalized contrast-enhanced MRI intensities onto 3D IA models reconstructed from MRA. We used expert-labelled enhancing regions to identify a threshold in a normalized contrast-enhanced MRI, which can best distinguish the regions of VWE, and validated this threshold by testing its accuracy in identifying enhancing regions in an independent dataset. Furthermore, we quantified the “enhancement area ratio” (EAR, the ratio of the enhancing area to the IA surface-area) and tested if it could delineate high-risk IA lesions. 

## 2. Methods

### 2.1. Patient Population

This retrospective study was approved by the institutional review board at the University at Buffalo (study 00004370). Patient consent was waived for this study. We retrospectively collected consecutive de-identified MRI images, and medical history from patients undergoing vessel wall MRI for IA at Dent Neurologic Institute between September 2019 and July 2020. Aneurysms of the cavernous segment of the ICA were excluded, because the high signal intensity of the cavernous sinus on the contrast-enhanced images prevented accurate identification and delineation of the aneurysm wall. 

### 2.2. Image Acquisition

Magnetic Resonance Imaging was performed on a 3T MRI scanner (Ingenia Elition 3.0 X, Philips Healthcare, Amsterdam, Netherlands) with a 16-channel head coil. The imaging protocol started with a non-enhanced 3D Time-of-Flight (TOF) MR angiography with the following parameters: axial plane, FOV = 20 × 20 cm, matrix = 324 × 282, slice thickness = 1.1 mm, slice overlap = 0.5 mm, TR = 25 ms, TE = 3.5 ms, Flip Angle = 25°, Compressed SENSE factor = 2.7, number of slices = 160, scan time = 6:05 min. This was followed by a non-enhanced 3D T1-weighted VISTA (Volume Isotropic Turbo Spin Echo Acquisition) black blood scan with the following parameters: axial plane, FOV = 18 × 18 cm, matrix = 360 × 358, slice thickness = 0.6 mm, slice overlap = 0.2 mm, reconstructed voxel size = 0.5 × 0.5 × 0.5 mm, TR = 450 ms, TE = 16.3 ms, Flip Angle = 90°, fat suppression = SPIR, Motion Sensitized Driven Equilibrium (MSDE) pre-pulse was applied to suppress venous flow, SENSE factor = 2 (RL), number of slices=105, scan time = 8:30 min. After this scan, a Gadolinium-based contrast agent was injected (gadobutrol, 0.1 mL/kg) and with a 6–7 min delay, the 3D T1-weighted VISTA scan with the same parameters was repeated. 

### 2.3. VWE Quantification Pipeline

To visualize and quantify VWE, we started by reconstructing 3D models from MRA data in order to provide the surface on which the intensities would be mapped. The MRA image was segmented and cleaned to generate a surface file using VMTK (www.vmtk.org, accessed 20 January 2020), as previously described [12]. To obtain all images into the same coordinate system, the non-enhanced and contrast-enhanced MR images were registered onto the raw MRA image using an open-source platform, 3D Slicer, which implemented the BRAINSFit module (https://www.slicer.org, accessed 20 January 2020) for mutual-information rigid body transformation of the images. For the registration, we used the following parameters: rigid (degrees of freedom = 6), 1% of samples (percentage of sampled pixels), GeometryAlign (for transform initialization) and linear interpolation (to scale image size). 

Next, MR images were used to obtain the enhancement signal intensities around the aneurysm surface. To reduce variations in MRI signal intensities between different cases, we normalized the post contrast images with the average intensity at the pituitary stalk (as it resides outside the blood–brain barrier) [13]. This approach has been shown to exhibit stable enhancement across different patients and different scanners of each respective case [14]. For each case, 5 different points on the pituitary stalk were randomly sampled from the sagittal plane of the contrast-enhanced MR image. The average value of these points was used for normalization. All of the cases had intact and normal appearing pituitary gland and stalk without structural abnormalities or abnormal enhancement.

**Figure 1:**
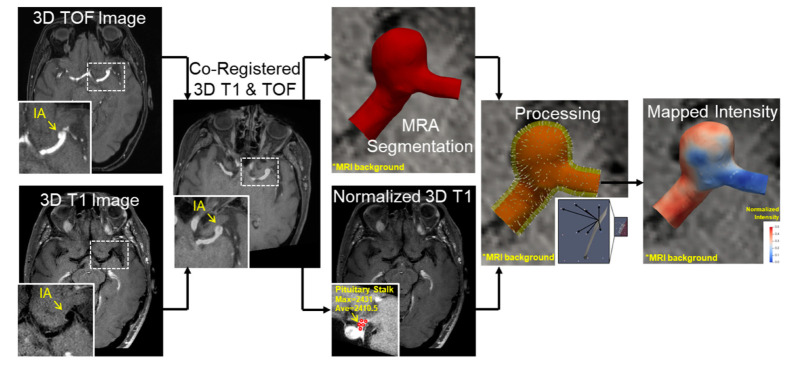
Pipeline for vessel wall enhancement quantification—Post-contrast MRI and MRA images are taken as inputs for this pipeline. The MR image is first normalized by the average contrast intensity at the pituitary stalk. The MR image is then registered on to the MRA image. The MRA image is segmented to generate a surface file. Following this, the region of interest is identified and then the voxels normal to each surface are interpolated on to the surface to generate the final contour as shown.

To map the surrounding MRI signal intensities onto the lumen surface, we developed an in-house MATLAB code (R2019a, The Mathworks, Natick, MA, USA). An inverse distance weighted interpolation technique was used to map the signal intensities onto the +surface, which is given by the following:

(1) $$I_{i}=\frac{\sum_{1}^{n}\frac{I_{n}}{d_{n}}}{\sum_{1}^{n}\frac{1}{d_{n}}}$$

where *I_i_* is the intensity at a point *i* on the aneurysm sac, n is the number of neighboring points in the registered MR image, *I_n_* is the intensity at a point *n* and *d_n_* is the Euclidean distance between point *i* and point *n*. For each point on the reconstructed IA surface, we chose the voxels in the co-registered MR image through which the normal passed, and used them for interpolation. The length of the normal for the IA was assumed based on the thickness of the parent vessel at the location of each aneurysm [15]. Hence, for aneurysms at the Internal Carotid Artery (ICA), Middle Cerebral Artery (MCA), Anterior cerebral artery (ACA) and basilar artery (BA, the length was assumed to be 0.66, 0.58, 0.45 and 0.61 mm, respectively. This was performed because the image resolution (0.5 × 0.5 × 0.5 mm) did not enable accurate measurement of the aneurysmal wall thickness, which typically ranges from 0.2 to 0.6 mm [16]. The entire workflow for VWE quantification is as shown in Figure 1.

As we used a constant length on the normal vector, it is possible that in regions where the IA wall is thin, the intensity of the surrounding brain tissue could be inadvertently mapped to the surface. For this reason, we used inverse distance weighted interpolation, which assures that the voxels closest to the surface would have the highest weightage when mapped. Using the maximum intensity instead of inverse distance weighted interpolation may result in an overestimation of intensities, e.g., the same voxels can be used by two neighboring faces, which results in duplication of values. This point is demonstrated in Supplementary Figure S1.

### 2.4. Identification of Enhancing Regions

For objective identification of enhancing regions, we sought to identify the optimal threshold of MRI intensity that can distinguish the enhancing and non-enhancing regions. To this end, a neuroradiologist with 12 years of experience reviewed all images, then manually marked each enhancing voxel in the normalized contrast-enhanced MR image using the Segment Editor tool in 3D Slicer to create a 3D “mask”. The marked voxels were then converted to a point cloud and the nearest faces on the 3D IA surface file that corresponded to the marked regions were identified.

To objectively identify and evaluate the optimal intensity threshold to delineate VWE, we randomly divided the cases into a training dataset and a validation dataset using a 70:30 split, and maintaining equal proportions of enhancing and non-enhancing cases (as defined by the radiologist). In the training dataset, we determined the optimal intensity threshold (T_0_) that can best delineate the enhancement regions from the non-enhancing regions using ROC (Receiver Operating Characteristic) analysis. Each point on each case was either classified as enhancing or non-enhancing by the experienced radiologist. Using this as the binary label and the mapped intensity as the continuous variable, we plotted an ROC curve. From this, the optimal threshold to define enhancement was determined by calculating the maximum Youden’s J index (i.e., maximum sensitivity and specificity).

Next, we validated the ability of the threshold to identify enhancing voxels in the testing dataset. In brief, all the IA surface voxel points from the testing cases were classified as enhancing or not based on the threshold T_0_, and were marked in a surface mask. The agreement of this mask with that marked by the radiologist was assessed across all testing cases via accuracy, sensitivity and specificity. Furthermore, in all enhancing cases noted by the radiologist, we calculated the Dice similarity coefficient (DSC), to measure the overlap in the surface map data, as follows:

(2) $$DSC=\frac{2*A\cap B}{A+B}$$

where *A* is the set of points marked in the mask, *B* is the set of points with an intensity greater than T_0_ and *A*∩*B* is the overlap of the points in both sets.

### 2.5. Enhancement Area Ratio as an Indicator of Risk

In the clinical setting, aneurysm VWE is assessed as a potential metric of IA rupture risk. Therefore, we sought to determine if the parameter enhancement area ratio (EAR)—defined as the ratio of the area of the sac greater than T_0_ to the total area of the sac—could delineate high-risk aneurysm cases. To do this, we calculated EAR for all cases and graded the risk of each IA using the 5-year rupture risk, defined by the ISUIA study, which is based on aneurysm size, location and history of rupture [17]. Additionally, we compared EARs performance to that of another VWE parameter, namely the aneurysm-to-pituitary stalk contrast ratio (CR_stalk_) [14], which is the maximum intensity in pituitary stalk-normalized VWE across the IA. CR_stalk_ has been shown to delineate unstable (or larger) IAs from stable (smaller) IAs with a sensitivity = 81.5%. In addition to ROC analysis, we also performed univariate analyses. Shapiro–Wilk tests were used to test if variables were normally distributed. If the variables were normally distributed, Student’s t-test was used, if not, a non-parametric Mann–Whitney U test was used. All the univariate analysis was performed using scipy packages in python [18]. For all statistics, a *p*-value < 0.05 was considered significant.

## 3. Results

### 3.1. Patient Characteristics

A total of 41 unruptured aneurysms from 38 patients were included in this study. The mean age of the patients was 68.4 ± 12.72 years, and 32 (84%) were female, 13 (34%) were smokers, 16 (42%) had hypertension and 5 (13%) had a prior history of subarachnoid hemorrhage. All the studied IAs were saccular and had an average size of 3.6 ± 2.7 mm. The majority of the aneurysms (41%) were located on the ICA, while 13 (32%) were at the MCA, 4 (10%) were at the PCom, 5 (12%) were at the ACA and 1 (2%) was at the tip of the basilar artery and 1 (2%) was at the Posterior Cerebral Artery. The average size of the whole cohort was 3.6 ± 2.7 mm.

Using our dataset of MRA and MR images from these patients, we created a pipeline for the quantification and 3D visualization of VWE (Figure 1), which was successfully implemented in all cases. On average, the time it took to analyze each aneurysm via this method was ~15 min. The resulting image is a fully interactive IA geometry with mapped VWE intensities that can be further analyzed, dichotomized and classified as needed by the user (exemplified in the subsequent sections). 

### 3.2. Determining Optimal Intensity Threshold

**Figure 2:**
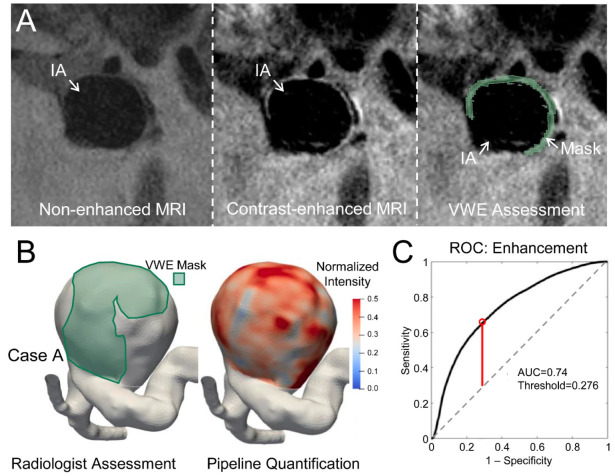
Identifying optimal threshold in the training dataset. (**A**). A cross-sectional slice of non-enhanced and contrast-enhanced MR images and the corresponding marked mask of a representative ICA case (Case A). (**B**). Reconstructed mask (marked in green) and the mapped intensities. (**C**). An ROC curve of the cumulative training set. The red line represents the location of maximal Youden’s J index.

To find an optimal intensity threshold by which to define enhancement, a training dataset of 30 IAs (see Supplementary Table S1 for patient characteristics of this dataset) was analyzed by a neuroradiologist, who marked the enhancing voxels on the aneurysm wall across all the cases (as shown in Figure 2A). Based on this assessment, 16 cases were found to have at least some voxels exhibiting enhancement, while 14 did not have any enhancing voxels. From the ROC analysis, the area under the ROC curve (AUC) for predicting the enhancement from voxel intensity was 0.74 (Figure 2B). Youden’s J index demonstrated that a normalized intensity of 0.276 best delineated the enhancing from the non-enhancing regions (Figure 2C). 

### 3.3. Validation of the Intensity Threshold

**Figure 3:**
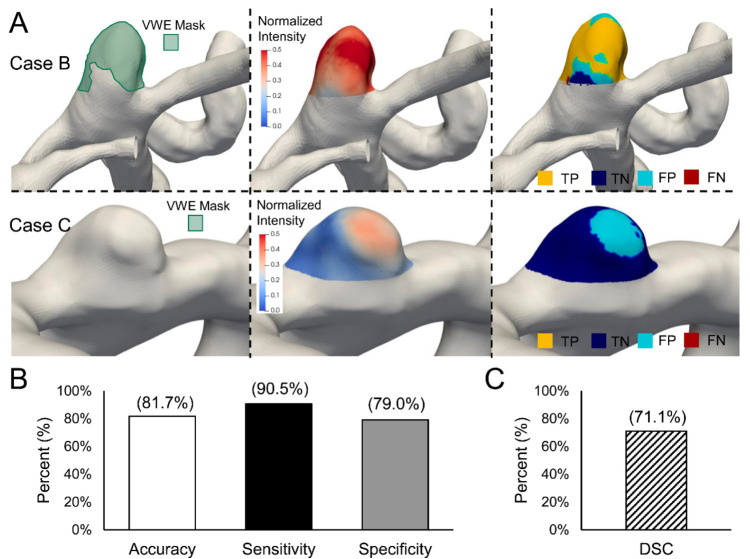
Results of VWE detection in the testing dataset. (**A**). Masks, mapped intensities and testing plots of 2 representative cases. Case B had a mask and Case C did not. The yellow, blue, cyan and red represent true positive (TP), true negative (TN), false positive (FP) and false negative (FN) classification of faces based on clinician marked voxels. Ideal case would have no FP and FN voxels. (**B**). Cumulative accuracy, sensitivity and specificity of all the faces in all the cases. (**C**). Average dice similarity coefficient (DSC) of only the cases with marked masks (*n* = 4) in the testing cohort.

To determine if the identified threshold could predict enhancing regions, we tested it in the validation cohort of 11 radiologist-marked IAs (see Supplementary Table S2 for patient characteristics of this dataset). In this cohort, four of the IAs were found to have some, while seven did not have any enhancing voxels. Two example predictions are shown in Figure 3A. The images on the left show the enhancing regions marked by the radiologist in green (Case B has VWE, whereas Case C does not), while the middle images show the visualization of enhancement intensity using our analysis pipeline. After thresholding, the areas/voxels of agreement (true positives and true negatives) and of disagreement (false positives and false negatives) are visualized in the images on the right. From the classifications across all cases, the accuracy was 81.7%, the sensitivity was 90.5% and the specificity was 79.0%. For the four IAs that were identified as cases with enhancement by the radiologist, the average DSC of the overlapping VWE areas was 71.1%, demonstrating good agreement. Of the seven cases assessed to not have any enhancement, the threshold determined that two IAs did have VWE, albeit at a low rate (one had 3% of its surface area classified as VWE, and the other had 11%).

### 3.4. CR_stalk_ and Enhancement Area Ratio as Indicators of Aneurysm Risk

**Figure 4:**
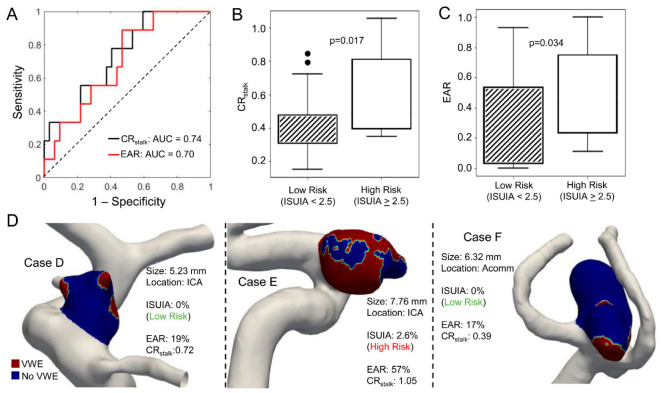
The EAR parameter can identify high-risk IAs. (**A**). An ROC curve of EAR and CR_stalk_ using IUSIA score ≥2.5 as the risk surrogate (**B**). Univariate analysis of CR_stalk_ in low and high-risk cases. (**C**). Univariate analysis of EAR in low and high-risk cases. (**D**). Representative cases and a contour plot demonstrating EAR.

Based on the ISUIA study, we determined an IA to be high risk if it had a 5-year rupture risk ≥2.5%. Using this threshold, nine aneurysms in our database were high-risk. In Figure 4, the ROC analysis revealed that the area under the curve (AUC) for the EAR was 0.7 and the AUC for the CR_stalk_ was 0.74 (Figure 4A). Based on this analysis, we found that the optimal EAR threshold for delineating high and low risk aneurysms was 23%, e.g., if an aneurysm had more than 23% of its area with an intensity greater than T_0_ (where T_0_ = 0.276), it would be considered high-risk. Univariate analyses showed that both the EAR and the CR_stalk_ are statistically significantly different between low- and high-risk IAs (EAR: *p* = 0.034, CR_stalk_: *p* = 0.017) (Figure 4B,C). This is highlighted by the three representative cases for low- and high-risk aneurysms in Figure 4D.

## 4. Discussion

Aneurysm wall contrast enhancement in MRI is a potential clinical correlate of IA instability and rupture [2,3,11]. In current clinical practice, aneurysmal wall enhancement is assessed qualitatively by manually comparing pre- and post-gadolinium MR images. However, to use the enhancement features as a tool for the risk stratification of IAs, an objective method to assess VWE that can be validated across different cases, scanners and centers is needed. In this study, we developed a semi-automated pipeline for VWE visualization and quantification, via the direct mapping of pituitary-stalk normalized contrast-enhanced MRI data onto segmented IA geometries (from MRA), followed by automated thresholding to objectively define regions of enhancement. Using expert assessments as the ground truth, our method was >80% accurate in identifying regions of enhancement in an independent testing dataset. Furthermore, the EAR showed promise as a binary classifier of whether an IA is high-risk or not.

Several pipelines have been proposed to standardize and quantify VWE in IAs [4,19,20,21]. One of the first attempts was made by Omodaka et al. [20], who, after normalization using pituitary stalk intensity, measured the maximal wall enhancement index by manually tracing voxel intensity on vessel lumen on the MR image. Wang et al. [21] also introduced a methodology where they manually measured the signal intensities at the neck, body, dome on four sequences and then utilized the maximum signal intensity to quantify VWE. While these metrics were shown to be associated with aneurysm rupture/instability in previous studies [3,22], they may lack objectivity and reproducibility. The manual marking and tracing of enhancing regions is often difficult and requires an expert to visually determine precise areas, which can be subject to errors. In our pipeline, the semi-automated mapping of signal intensity patterns on the 3D view of the aneurysm and objective delineation of regions with enhancement avoids such subjectivity and human error.

Additionally, the previous methodologies for measuring VWE in IA all quantified a single-value metric for the entire aneurysm, which, itself, cannot convey signal intensity variation throughout the wall. Indeed, IAs might demonstrate circumferential or focal enhancement features, which may have clinical significance. For example, studies have shown that focal enhancement may be indicative of the presence of an intraluminal thrombus [23]. The use of a 3D segmented MRA in our pipeline enabled the direct mapping of the VWE metric for an objective identification of the enhancement pattern and a complete 3D view of the aneurysm. To our knowledge, only one other study rendered a 3D visualization of the aneurysmal VWE [10]. Khan et al. mapped MRI intensities on landmark-based registered digital subtraction angiography IA segmentations. While the methodology presented in this study utilizes a similar procedure for visualization, it goes further by objectively delineating regions with enhancement and categorizing the IA as enhancing or non-enhancing. Thus, our pipeline may be a promising starting point for developing more robust stratification metrics of aneurysmal VWE, as it visualizes the wall enhancement in 3D space, enabling the easy delineation of enhancement patters, i.e., focal or circumferential enhancement. Although the agreement between masks defined by the neuroradiologist and that obtained through our pipeline is moderate (DICE coefficient: 71%), it can nevertheless be improved. The use of a larger cohort of images from different centers combined with input from multiple experienced users in the field is warranted for a more robust threshold. This would increase the utility of this pipeline since the collective experience of the users would reduce bias and subjectivity, resulting in a more reliable metric.

Clinically, unstable and rupture-prone aneurysms have been shown to demonstrate enhancement features compared to those that are low-risk and remain stable. As a preliminary metric that considers both the presence of enhancement and its relation to the area of the IA sac, we calculated the EAR. This is similar to the manually calculated percent enhancement described by Larsen et al. [11], which was found to be significant positively correlated to the IA risk metric, PHASES (population-hypertension-age-size-earlier SAH-site) score. The EAR may be a useful parameter because it normalizes the enhancing area by the IA surface area, thus minimizing the influence of small high-intensity image artifacts that may be present [24]. Based on our ROC analysis, the EAR was able to correctly identify clinically high-risk IAs with an AUC of 0.70, albeit an independent validation in a separate cohort is still needed. This was similar to the performance of the CR_stalk_, which had an AUC of 0.74. Furthermore, based on evaluation of enhancement by three clinicians, the EAR and CR_stalk_ also reflected the clinical categorization of IA, i.e., binary assessment of enhancing or not (CR_stalk_: 0.741 and EAR: 0.740) (Details provided in Supplementary Figure S2). We recognize, however, that metrics such as the EAR and CR_stalk_ are only single-values, and do not consider enhancement patterns. We postulate that more complex metrics that consider both the intensity and pattern of enhancement will be needed for a more robust assessment of IA instability/rupture risk.

This study has several limitations. First, the small sample size limits the confidence in the robustness of the optimal enhancement threshold. Future studies in larger cohorts are required. Second, input from one expert neuroradiologist at a single center was considered as the benchmark in VWE identification. Multiple raters of data from multiple institutions are needed to limit subjectivity. Third, for mapping the signal intensities onto the luminal surface, we assumed the thickness of the aneurysm wall to be homogeneous and that of the thickness of the parent artery. However, previous literature has shown that the wall composition and thickness has variations and is unique in each IA [25]. A higher resolution MRI could be implemented to accurately capture the IA wall thickness and prescribe vector lengths. A previous study has demonstrated a just-enough-interaction method for segmenting the IA wall, which could lead to a better approximation of wall thickness [26]. Finally, we also acknowledge that the EAR threshold is also dependent on the threshold of the ISUIA score used on the cohort, which is subjective.

## 5. Conclusions

In this study we demonstrated a pipeline for the 3D visualization and quantitation of aneurysmal VWE from non-invasive MR imaging. In doing so, we defined an objective threshold of vessel wall contrast enhancement (T_0_ = 0.276) based on input from a neuroradiologist. We also demonstrated the potential usage of the EAR as a metric for the classification of high-risk aneurysms with a threshold of 23%. We hope this could contribute to the development and clinical utilization of quantification techniques for the assessment of vessel wall enhancement.

## Data Availability

The data presented in this study are available on request from the corresponding author. The data are not publicly available due to continued analysis by the corresponding author’s research team and IRB restrictions.

## Supplementary Materials

The following are available online at www.mdpi.com/article/10.3390/diagnostics11101742/s1, Figure S1: Sensitivity analysis of mapping technique, Figure S2: Clinical evaluations of IAs, Table S1: Patient characteristics for training dataset, Table S2: Patient characteristics for testing dataset.
