# Supplementary material for: An Image-Based Workflow for Objective Vessel Wall Enhancement Quantification in Intracranial Aneurysms
Source: Diagnostics (Basel). 2021 Sep 22;11(10):1742. doi: 10.3390/diagnostics11101742 (PMC8534502; doi:10.3390/diagnostics11101742)
Supplement: Supplementary file 1 [file diagnostics-11-01742-s001.zip › diagnostics-1299807-supplementary.pdf]

Supplemental Information for: An Image-Based Workflow for Objective Vessel Wall Enhancement Quantification in Intracranial Aneurysms

Sricharan S. Veeturi [1,2], Nandor Pinter [3,6], Andre Monteiro [3], Ammad Baig [3], Hamid Rai [3], Muhammad Waqas [1,3], Adnan H. Siddiqui [1,3], Hamidreza Rajabzadeh-Oghaz [1,3], *Vincent M. Tutino [1,2,3,4,5]

Affiliations: [1]Canon Stroke and Vascular Research Center; [2]Department of Mechanical and Aerospace Engineering, [3]Department of Neurosurgery, [4]Department of Biomedical Engineering, [5]Department of Pathology and Anatomical Sciences, University at Buffalo, Buffalo, NY, USA; [6]Dent Neurologic Institute, Buffalo, NY, USA

Email Addresses: sanyasis@buffalo.edu, npinter@dentinstitute.com, amonteiro@ubns.com, abaig@ubns.com, hrai@ubns.com, mwaqas@ubns.com, asiddiqui@ubns.com, hrajabza@buffalo.edu, vincentt@buffalo.edu

***Corresponding author**:
Vincent Tutino, PhD
Canon Stroke and Vascular Research Center
Clinical and Translational Research Center
875 Ellicott Street
Buffalo, NY 14214 USA
E-mail: vincentt@buffalo.edu; Phone: (716) 829-5400
Fax: (716) 854-1850

Supplemental Tables

Supplementary Table S1: Patient characteristics for training dataset. *

Characteristic	Value
Age	66.9 ±15.0 years
Female gender	22/27 (81.5%)
Smoking	9/27 (33.3%)
Hypertension	10/27 (37.0 %)
Family history of IA	3/27 (11.1 %)
Patients with Multiple IAs	3/27 (11.1 %)
IA Location	
PCom	4/30 (13.3%)
ACom	5/30 (16.7%)
ICA	17/30 (56.7 %)
MCA	13/30 (43.3%)
BA	2/30 (6.7%)
IA size (max. height)	3.35±1.8 mm

* (Abbreviations: IA= Intracranial Aneurysm, PCom= Posterior communicating artery, ACom= Anterior communicating artery, ICA= Internal carotid artery, MCA= Middle cerebral artery, BA= Basilar artery, max.= maximum)

Supplementary Table S2: Patient characteristics for testing dataset. *

Characteristic	Value
Age	72.1±10.2 years
Female gender	10/11 (90.9%)
Smoking	4/11 (36.4%)
Hypertension	6/11 (54.5 %)
Family history of IA	2/11 (18.2 %)
Patients with Multiple IAs	0/11 (0 %)
IA Location	
PCom	1/11 (9.1%)
ACom	3/11 (27.3%)
ICA	2/11 (18.2 %)
MCA	4/11 (36.4%)
BA	1/11 (9.1%)
IA size (max. height)	4.2±4.5 mm

* (Abbreviations: IA= Intracranial Aneurysm, PCom= Posterior communicating artery, ACom= Anterior communicating artery, ICA= Internal carotid artery, MCA= Middle cerebral artery, BA= Basilar artery, max.= maximum)

Supplemental Figures

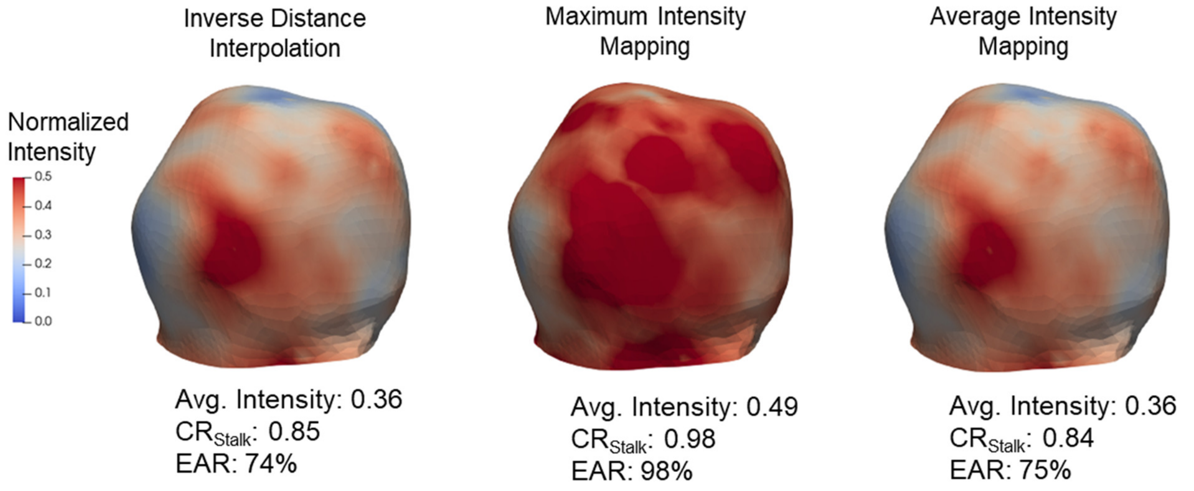

Supplementary Figure S1. Sensitivity analysis of mapping technique. Instead of using inverse distance weighted interpolation, we used the maximum intensity of selected voxels along the normal and also use the average. We observed there was a large change in all the VWE quantification metrics between the IDW interpolation and maximum intensity mapping. This is because based on the curvature, neighboring faces on the sac surface choose the same voxel intensity and map it onto the surface. The case shown above has 30% of the faces with the same intensity value as the neighboring face. However, we do not see any noticeable difference between inverse distance weighted interpolation mapping and average intensity mapping (Average intensity of all selected voxels).

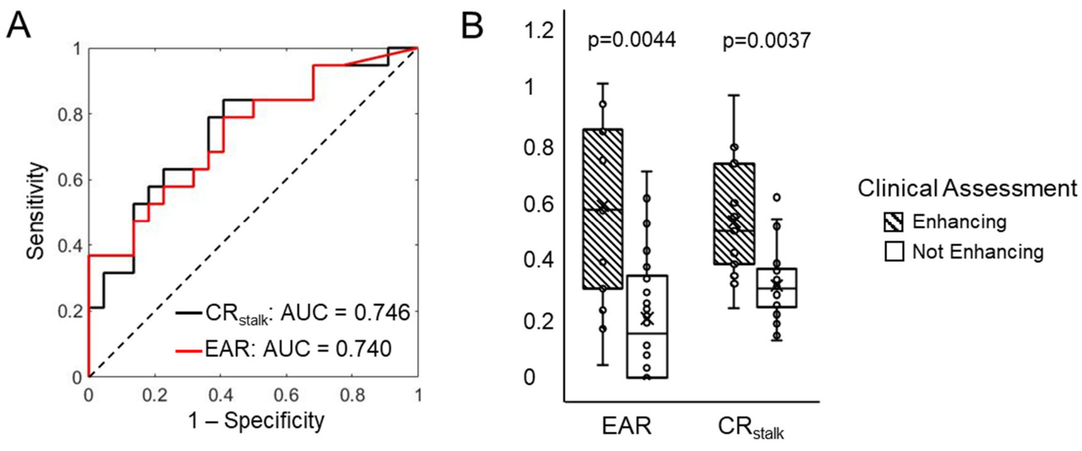

Supplementary Figure S2: Clinical evaluations of IAs. All the 41 cases present in this study were classified as enhancing or non-enhancing by 3 clinicians using both non-enhanced and contrast-enhanced MRI images. A majority voting system was used to then determine if each case had enhancement or not. We then used these binary labels to evaluate the performance of CR_{stalk} as well as EAR in delineating enhancing and non-enhancing aneurysms. **A).** The ROC curve shows CR_{stalk} has an AUC=0.75 and EAR 0.74. **B).** We also performed univariate analysis and found that EAR and CR_{stalk} are both significantly higher (EAR: p=0.004; CR_{stalk}: p=0.004) than in non-enhancing IAs.
